# Heat and mass transfer with entropy optimization in hybrid nanofluid using heat source and velocity slip: a Hamilton–Crosser approach

**DOI:** 10.1038/s41598-023-39176-5

**Published:** 2023-07-31

**Authors:** Sidra Afzal, Mubashir Qayyum, Gilbert Chambashi

**Affiliations:** 1 National University of Computer and Emerging Sciences FAST Lahore Campus, Lahore, Pakistan; 2 School of Business Studies, Unicaf University, Longacres, Lusaka, Zambia

**Keywords:** Fluid dynamics, Nanoparticles

## Abstract

The modeling and analysis of hybrid nanofluid has much importance in industrial sector where entropy optimization is the key factor in different processes. This mechanism is also used in medical industry, where it can be used for separation of blood cells (red and white blood cells, platelets and plasma) by centrifuge process, treating cancers, and drug transport. In light of this importance, current study is focused on mathematical modeling and analysis of blood based hybrid nanofluid between rotating disks with various shapes of nanoparticles. The shape factors are taken into account with Hamilton–Crosser model as spherical, brick, cylinder and platelet in the current scenario, with special reference to entropy optimization. In order to solve modeled nonlinear and non-dimensional system, optimal homotopy analysis approach is utilized through Wolfram MATHEMATICA 11.3 software. Error estimation and convergence analysis confirms that obtained semi-analytical solutions are valid and reliable. Velocity, temperature and concentration profiles are analyzed against important fluid parameters. Fluid velocity decreased in all directions when unsteady parameter $\mathbb {B}$ and Darcy number *Da* increased while the slip parameters $\zeta _{1}$ and $\zeta _{2}$ decreased the nanofluid velocity. It is observed that in case of brick shaped nanoparticles, fluid temperature is enhanced as compared to other shape factors in the study. Minimal entropy generation is captured in case of spherical nanoparticles, while highest heat transfer is observed in platelet shaped nanoparticles. Furthermore, numerical optimization of entropy is performed against different values of $\hbar$ and volume fractions $\varphi _{Rd}$ and $\varphi _{Al}$. Minimized entropy is recovered to be zero when $\hbar =-0.6$, $\varphi _{Rd}=2\%$ and $\varphi _{Al}=1\%$.

## Introduction

Hybrid nanofluids are colloidal mixtures containing two type of nanoparticles mixed in a single base fluid. These fluids are useful in applications where heat and mass transfer enhancement is required to obtain more efficient and effective systems. Hybrid nanofluids especially with different shapes of nanoparticles can further improve the heat transfer effects due to which their study has gained interest of many researchers. Kashi’e et al.^1^ investigated the effects of shape factor in nanofluid on Riga plate. Subray et al.^2^ comparatively analyzed the flow of a nano and hybrid nanofluid for brick, blade and laminar shaped nanoparticles. Li and You^3^ simulated the flow of a water-based hybrid nanofluid over a stretching sheet with various shapes of copper and alumina nanoparticles. Akbar et al.^4^ studied the Maxwell nanofluid flow over a linearly stretched surface. Analysis on cross flow of hybrid nanofluid with numerous nanoparticle shapes is performed by Ramesh^5^. Study on a rate type nanofluid over a magnetized stretching sheet is performed by Liu et al.^6^. Dinarvand and Rostami^7^ studied squeezing of hybrid nanofluid with variable shapes. Ghobadi and Hassankolaei^8^ numerically simulated the hybrid nanofluid on cylinder with different shape factors. Chung et al.^9^ analyzed three dimensional hybrid nanofluid flow with heat source/sink. Gholinia et al.^10^ explored nanofluid with varying shapes of titanium oxide and alumina nanoparticles. Nasir et al.^11^ studied hybrid nanofluid flow over a Darcy-Forchheimer porous surface. Waqas et al.^12^ explored different shapes of gold nanoparticles in Sisko fluid. Li et al.^13^ studied slip effects on a nanofluid flow over stretching sheet.

Increase in energy generation gathered much attention in last decade. Bhatti et al.^14^ investigated Williamson nanofluid with swimming gyrotactic microorganisms. Khalaf et al.^15^ improved the heat transfer effects in a nanofluid with porous media. Chu et al.^16^ studied heat transfer of a hybrid nanofluid in a microchannel. Ahmad et al.^17^ analyzed the bio-convective flow of a gyrotactic microbes based nanofluid flow over a non-linearly stretched sheet and passing through a porous medium. Muhammad et al.^18^ investigated the Darcy-Forchheimer porous medium flow of a carbon nanotubes based nanofluid. Li et al.^19^ enhanced the heat transfer properties of the time-dependent viscous fluid flow. Gul et al.^20^ analyzed heat transfer in a hybrid nanofluid flow in a porous chamber. Panigrahi et al.^21^ numerically simulated the effects of porous media on MHD flow of a Casson nanofluid using Runge-Kutta method with shooting technique. Babu et al.^22^ simulated the heat and mass transfer effects in a nanofluid flow over a wedge. Nasir et al.^23^ enhanced the heat transport properties in stagnation point flow of a hybrid nanofluid. Esfe et al.^24^ studied the impact of porous medium on three different types of convective transfer through heat. Recently, Prasannakumara^25^ investigated the influence of porous media on methanol and *NaAlg* based nanofluid flow through Tiwari-Das model. Ragupathi et al. in^26^ explored radiative Casson nanofluid over a radially stretching and rotating disk.

Many applications of rotating disks involve heat generation and absorption phenomena in order to perform the task optimally. It can either require higher temperatures or extremely lower temperatures depending on the phenomena under consideration. For instance, in order to separate platelets and other components from blood rotation, an ambient temperature must be maintained in order to achieve the desired results. Different studies in literature have taken heat source/sink into account. Ali et al.^27^ sought out to improve the thermal transport of two types of nanofluids (mono and hybrid) passing over an inclined sheet with heat source/sink effects. Nasir et al.^28^analyzed entropy generation in ethylene glycol and water based nanofluid. Yaseen et al.^29^ investigated the flow of a water based hybrid nanofluid past a moving convective heated surface with heat source/sink, velocity slip and non-linear thermal radiation. Sajid et al. in^30^ investigated a Cross non-Newtonian tetra hybrid nanofluid flow in a stenosed artery. Sulochana and Kumar^31^ enhanced the rate of of heat transfer with heat source and sink in a mono and hybrid nanofluid over a stretching surface. Chu et al.^32^ analyzed Jeffrey nanofluid with chemical reaction between two disks. Chamkha et al.^33^ numerically analyzed the copper-alumina hybrid nanofluid flow with water as base fluid inside a partially heated square cavity under heat generation and absorption effects. Gorla et al.^34^ investigated heat source and sink effects on a hybrid nanofluid flow in a porous cavity. In a recent study, Yasir et al.^35^ applied a non-uniform heat source/sink in an ethylene glycol based hybrid nanofluid with Hamilton-Crosser model. Saleh et al.^36^ simulated effects of heat generation and absorption on a Maxwell hybrid nanofluid with MHD effects. Dinarvand et al.^37^ performed a numerical investigation on squeezing flow of a water based hybrid nanofluid between two collateral sheets influenced by heat generation and absorption.

Entropy generation is the useful energy dissipated in the environment and it results in reduced efficiency of engineering systems and biological processes. Many studies in recent years are focused on entropy minimization to provide best possible conditions and obtain maximum output as a result. Li et al.^38^ attempted to minimize the entropy generation in membrane reactor of methanol synthesis with various geometries by using optimal control theory and linear programming. Khan et al.^39^ investigated the entropy minimization in a non-linear thermal radiative flow of hybrid nanofluid with water as a base fluid. Obalalu et al.^40^ minimized the entropy generation in a Casson nanofluid flow over a stretching Riga plate and non-Darcy porous medium. Nasir et al.^41^ optimized entropy generation in a Maxwell nanofluid flow. Li et al.^42^ simulated entropy generation in stagnation point flow of Carreau nanofluid. Munawar et al. in^43^ investigated the entropy minimization of a hybrid nanofluid flow inside a corrugated triangular annulus with magnetic effects and free convection. Khan et al.^44^ simulated entropy generation in a viscous nanofluid with second order velocity slip. Ibrahim et al.^45^ analyzed entropy generation in a nanofluid flow with twisted porous objects. Mabood et al.^46^ minimized the entropy generation in a Jeffery nanofluid boundary layer flow over a permeable stretching sheet with non-linear thermal radiation and activation energy. Acharya et al.^47^ investigated the entropy generation in a ferrous oxide and graphene oxide hybrid nanofluid over an unsteady spinning disk with slip effects.

The focus of current study is entropy analysis, and modeling of heat and mass transfer in a blood based unsteady hybrid nanofluid with radium and alumina nanoparticles having various shapes including spherical, brick, platelet and cylindrical through Hamilton-Crosser model. The nanoparticles of current study are important in enhancing heat and mass transfer properties of blood which is useful in many applications of medical industry including drug transport, cancer treatment and centrifuging blood to obtain its components (platelets, red and white blood cells). The flow is simulated with slip boundary conditions and fluid rotation between double rotating disks. The flow is also influenced by magnetic field, porous medium and heat sink/source. Using appropriate transforms modeled equations are converted to system of nonlinear ODEs. The solution method adopted is a semi-analytical approach namely, homotopy analysis method (HAM). The series form solution obtained with this method are validated through mean square errors and convergence table. Moreover, solutions obtained through HAM are also compared with Runge-Kutta 4th order solutions to provide further validation of results. The nanofluid flow in radial axial and tangential directions is graphically analyzed. Fluid temperature and concentration is simulated against pertinent fluid parameters. Entropy generation is presented numerically and graphically. In rest of the article, mathematical formulation is given in Section “Mathematical Formulation,” solution methodology is presented in Section “Proposed Methodology with Convergence Analysis,” results are simulated graphically and discussed in Section “Results and Discussion” and finally major conclusions drawn from this study are given in Section “Conclusion”.

## Mathematical formulation

**Figure 1:**
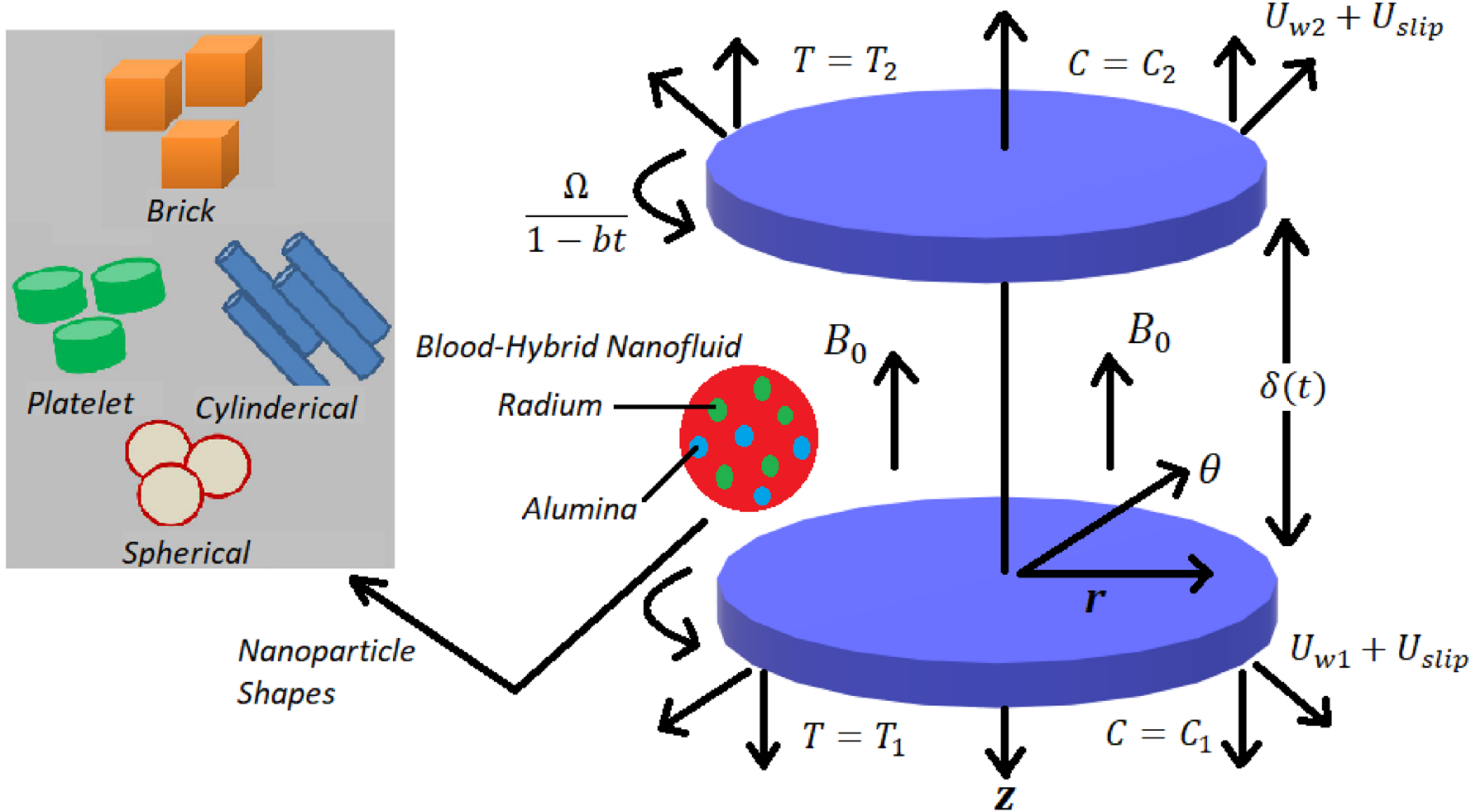
Blood Flow Geometry.

The flow geometry consists of double rotating disks with cylindrical coordinated $(r,\theta ,z)$ and blood based hybrid nanofluid which contains Radium *Rd* and alumina $Al_2O_3$ nanoparticles. The fluid is immersed in a porous media with its flow influenced by magnetic field acting along z-axis. The disks are rotating and stretching with velocity slip acting in r-direction. The temperature and concentration at lower and upper disks are $T_{1}, T_{2}$ and $C_{1}, C_{2}$ respectively. Moreover, Heat source/sink is also applied on the fluid. Detailed geometry of this flow problem is presented in Fig. 1. The governing equations are given below^48^

1 $$\begin{aligned}{} & {} \frac{\partial u}{\partial r}+\frac{\partial w}{\partial z}+\frac{u}{r}= 0, \end{aligned}$$

2 $$\begin{aligned}{} & {} \frac{\partial u}{\partial t}+u\frac{\partial u}{\partial r}+w\frac{\partial u}{\partial z}-\nu _{hnf}\nabla ^{2}u-\frac{v^{2}}{r}+\frac{\sigma _{hnf}B^{2}_{0}}{\rho _{hnf}}u+\frac{\mu _{hnf}\varkappa ^{*}}{\rho _{hnf}k_{0}}u=0, \end{aligned}$$

3 $$\begin{aligned}{} & {} \frac{\partial v}{\partial t}+u\frac{\partial v}{\partial r}+w\frac{\partial v}{\partial z} -\nu _{hnf}\nabla ^{2}v+\frac{uv}{r}+\frac{\sigma _{hnf}B^{2}_{0}}{\rho _{hnf}}v=0,\end{aligned}$$

4 $$\begin{aligned}{} & {} \frac{\partial T}{\partial t}+u\frac{\partial T}{\partial r}+w\frac{\partial T}{\partial z} = \frac{k_{hnf}}{(\rho Cp)_{hnf}}\nabla ^{2}T+\frac{Q_{s}}{(\rho C_{p})_{hnf}}(T-T_{\infty }), \end{aligned}$$

5 $$\begin{aligned}{} & {} \frac{\partial C}{\partial t}+u\frac{\partial C}{\partial r}+w\frac{\partial C}{\partial z} =D_{hnf} \frac{\partial ^{2} C}{\partial z^{2}}, \end{aligned}$$

with following conditions on the boundary

6 $$\begin{aligned} \begin{aligned} u=U_{w1}(r,t)+U_{slip}(r,t),\quad v=\frac{r \Omega }{1-bt},\quad w=0,\quad T=T_{1},\quad C=C_{1}\quad at\quad z=0\\ u=U_{w2}(r,t),\quad v=0,\quad T=T_{2},\quad C=C_{2}\quad at\quad z=\delta (t)=\sqrt{\frac{\nu _{f} }{\Omega }(1-bt)}\\ U_{w1}(r,t)=\frac{q_{1}r}{1-bt},\quad U_{w2}(r,t)=\frac{q_{2}r}{1-bt},\quad U_{slip}(r,t)=L\nu _{f}\frac{\partial u}{\partial z},\quad L=N(1-bt)^{1/2} \end{aligned} \end{aligned}$$

where (*u*, *v*, *w*) are the velocities of fluid in *r*, $\phi$ and *z* directions, respectively. $\nu _{hnf}$, $\sigma _{hnf}$, $\rho _{hnf}$, $k_{hnf}$, $(\rho Cp)_{hnf}$ and $D_{hnf}$ are the viscosity, electrical conductivity, density, thermal conductivity, specific heat and thermal diffusivity of hybrid nanofluid. $B_{0}^{2}$ is the magnetic field strength, $Q_{s}$ is the heat source/sink, *T* is the temperature and *C* is the concentration of fluid. $U_{w1}$ and $U_{w2}$ are the velocities at both disks characterized by stretching, $U_{slip}$ is the slip velocity and *L* is the slip distance. $\Omega$ is the magnitude of disk rotation and $(1-bt)>0$ is non-dimensional with *t* having unit (*s*) and *b* having the unit $(s^{-1})$.

### Thermo-physical relations and properties

Behavior of nanofluids vary depending on base fluid and nanoparticles taken into account. There are many models in literature that characterize various properties of nanofluids. The hybrid nanofluid model considered in this study is the Hamilton-Crosser model^49^ which also considers the shape factor of the nanoparticles involved. The thermo-physical quantities in this case are given below

#### Thermal conductivity

The thermal conductivity for the current phenomena is^50^

7 $$\begin{aligned} \begin{aligned} k_{hnf}=k_{\phi }\left[ \frac{(n_{2}-1)k_{\phi }+k_{Al}-\varphi _{Al}(k_{\phi }-k_{Al})(n_{2}-1)}{(n_{2}-1)k_{\phi }+k_{Al}-\varphi _{Al}(k_{Al}-k_{bf})}\right] , \end{aligned} \end{aligned}$$

here the quantity $k_{\phi }$ is defined as

8 $$\begin{aligned} \begin{aligned} k_{\phi }=k_{f}\left[ \frac{(n_{1}-1)k_{f}+k_{Rd}-\varphi _{Rd}(k_{f}-k_{Rd})(n_{1}-1)}{(n_{1}-1)k_{f}+k_{Rd}-\varphi _{Rd}(k_{Rd}-k_{f})}\right] . \end{aligned} \end{aligned}$$

where $n_{1}$ and $n_{2}$ correspond to the shape constants of radium and alumina nanoparticles, respectively. In this study 4 different nanoparticle shapes i.e., spherical, brick, cylindrical and platelet shapes are considered. The shape constants in this regard are presented in Table 1. ’*hnf*’ presents the quantities of hybrid nanofluid,’*f*’ presents the quantities of base fluid whereas ’*Rd*’ and ’*Al*’ presents the radium and alumina quantities, respectively.

**Table 1:** Shape constants for various nanoparticle shapes^50^.

*n*	Shape
5.7	Platelet
4.9	Cylinder
3.7	Brick
3.0	Spherical

#### Thermal diffusivity

The hybrid nanofluid thermal diffusivity is given as^50^

9 $$\begin{aligned} \begin{aligned} D_{hnf}=D_{f}(1-\varphi _{hnf}), \end{aligned} \end{aligned}$$

the volume fraction $\varphi _{hnf}$ is

10 $$\begin{aligned} \varphi _{hnf}=\varphi _{Rd}+\varphi _{Al}. \end{aligned}$$

#### Electrical conductivity

The electrical conductivity of hybrid nanofluid containing radium and alumina nanoparticles is^50^

11 $$\begin{aligned} \begin{aligned} \frac{\sigma _{hnf}}{\sigma _{f}}=\frac{\varphi _{\sigma }+2k_{f}+2(\varphi _{Rd}\sigma _{Rd}+\varphi _{Al}\sigma _{Al})-2\varphi _{hnf}\sigma _{f}}{\varphi _{\sigma }+2k_{f}-(\varphi _{Rd}\sigma _{Rd}+\varphi _{Al}\sigma _{Al})+\varphi _{hnf}\sigma _{f}}, \end{aligned} \end{aligned}$$

where the quantity $\varphi _{\sigma }$ is given below

12 $$\begin{aligned} \begin{aligned} \varphi _{\sigma }=\frac{\varphi _{Rd}\sigma _{Rd}+\varphi _{Al}\sigma _{Al}}{\varphi _{hnf}} \end{aligned} \end{aligned}$$

#### Kinematic and dynamic viscosity

The kinematic viscosity is given as^50^

13 $$\begin{aligned} \begin{aligned} \nu _{hnf}=\frac{\mu _{hnf}}{\rho _{hnf}}, \end{aligned} \end{aligned}$$

and the dynamic viscosity is

14 $$\begin{aligned} \begin{aligned} \mu _{hnf}=\frac{\mu _{f}}{(1-\varphi _{Rd})^{5/2}(1-\varphi _{Al})^{5/2}}, \end{aligned} \end{aligned}$$

#### Density and Heat Capacity

The density and heat capacity of hybrid nanofluid is^50^

15 $$\begin{aligned} \begin{aligned} \rho _{hnf}=(1-\varphi _{hnf})\rho _{f}+\varphi _{Rd}\rho _{Rd}+\varphi _{Al}\rho _{Al}, \end{aligned} \end{aligned}$$

and

16 $$\begin{aligned} \begin{aligned} (\rho Cp)_{hnf}=(1-\varphi _{hnf})(\rho Cp)_{f}+\varphi _{Rd}(\rho Cp)_{Rd}+\varphi _{Al}(\rho Cp)_{Al}. \end{aligned} \end{aligned}$$

**Table 2:** Thermophysical properties of radium, alumina and blood^51,52^.

Physical properties	*Rd*	$Al_{2}O_{3}$	Blood
$$\rho$$(kgm$$^{-3})$$	5500	3970	1053
$$\sigma$$(Sm$$^{-1})$$	1$$\times 10^{6}$$	$$3.5\times 10^{7}$$	0.8
$$C_{p}$$(J kg$$^{-1}$$K$$^{-1})$$	0.12	765	3.617
*k*(Wm$$^{-1}$$K$$^{-1})$$	19	40	0.492

### Non-dimensional analysis

We non-dimensionalize the system of partial differential equations given in Eqs. (1)–(4) by introducing the following similarity transformations^53^

17 $$\begin{aligned} \begin{aligned} u=\frac{\Omega r}{1-bt} F'(\eta ),\quad v=\frac{\Omega r}{1-bt}G(\eta ),\quad w=-2\sqrt{\frac{\Omega \nu _{f}}{1-bt}}F(\eta ),\\ \eta =\frac{z}{(1-bt)^{1/2}}\sqrt{\frac{\Omega }{\nu _{f}}},\quad \phi (\eta )=\frac{C-C_{2}}{C_{1}-C_{2}},\quad \theta (\eta )=\frac{T-T_{2}}{T_{1}-T_{2}}, \\ \end{aligned} \end{aligned}$$

Use (17) in (1)-(4), we get

18 $$\begin{aligned}{} & {} \chi _{3}F'''-F'^{2}+2FF''+\frac{\chi _{1}}{\chi _{2}}MF'-\mathbb {U}\left( F'+\frac{\eta }{2}F''\right) -G^{2}+\chi _{3}\frac{F'}{Da}=0, \end{aligned}$$

19 $$\begin{aligned}{} & {} \chi _{2}\chi _{3}G''-\chi _{2}\mathbb {U}\left( G+\frac{\eta }{2}G'\right) -\chi _{1}MG-2\chi _{2}(GF'-FG')=0, \end{aligned}$$

20 $$\begin{aligned}{} & {} \chi _{4}\theta ''-Pr\chi _{5}(\mathbb {U}\eta -2F)\theta '+\frac{1}{\chi _{5}}H_{s}\theta =0, \end{aligned}$$

21 $$\begin{aligned}{} & {} \frac{\chi _{6}}{\mathcal {S}_c}\phi ''-\frac{\mathbb {U}\eta }{2}\phi '+2F\phi '=0, \end{aligned}$$

subject to

22 $$\begin{aligned} \begin{aligned}{}&\quad F(0)=0,\quad F'(0)=\zeta _{1}+\lambda F''(0),\quad \phi (0)=\theta (0)=G(0)= 1, when\quad \eta =0,\\&\quad F(1)=0,\quad F'(1)=\zeta _{2},\quad \theta (1)=\phi (1)=G(1)=0, when \quad \eta =1. \end{aligned} \end{aligned}$$

Where following are the dimensionless quantities in Eqs. (18)–(22) are

23 $$\begin{aligned} \begin{aligned}{}&\chi _{1}=\frac{\sigma _{hnf}}{\sigma _{f}},\quad \chi _{2}=\frac{\rho _{hnf}}{\rho _{f}},\quad \chi _{3}=\frac{\nu _{hnf}}{\nu _{f}},\quad \chi _{4}=\frac{k_{hnf}}{k_{f}},\quad \chi _{5}=\frac{(\rho Cp)_{hnf}}{\rho Cp_{f}}, \\&\chi _{6}=\frac{D_{hnf}}{D_{f}},\quad Da=\frac{\Omega \rho _{f}k_{0}}{\mu _{f}\varkappa ^{*}(1-bt)},\quad \lambda =N\sqrt{\Omega \nu _{f}},\quad \mathcal {S}_{c}=\frac{\nu _{f}}{D_{f}},\quad \mathbb {B}=\frac{b}{\Omega }, \\&Pr=\frac{(\rho Cp)_{f}\nu _{f}}{k_{f}},\quad H_{s}=\frac{Q_{s}(1-bt)}{(\rho C_{p})_{f}\Omega },\quad M=B_{0}^{2}\frac{\sigma _{f}}{\rho _{f}\Omega },\quad \zeta _{1}=\frac{q_{1}}{\Omega },\quad \zeta _{2}=\frac{q_{2}}{\Omega }. \end{aligned} \end{aligned}$$

here $\chi _{i}$ are the non-dimensional ratios of nanofluid quantities, *Da* is the Darcy number, $\lambda$ is the slip parameter, $\mathcal {S}_{c}$ is the Schmidt number, $\mathbb {B}$ is the unsteadiness parameter, *Pr* is the Prandtl number, $H_{s}$ is the heat source/sink parameter (where $H_{s}<0$ corresponds to heat sink and $H_{s}>0$ corresponds to heat source), *M* is the magnetic interaction parameter and $\zeta _1$ and $\zeta _{2}$ are the stretching parameters.

### Skin friction, heat and mass transfer

At the disk wall^53,54^

24 $$\begin{aligned} \begin{aligned}{}&\mathbb {C}_{f}=\frac{1}{\rho _{f}(\Omega r)^{2}}\sqrt{[\mu _{hnf}(u_{z}+u_{\phi })]_{z=0}^{2}+\left[ \mu _{hnf}\left( v_{z}+\frac{1}{r}+w_{\phi }\right) \right] _{z=0}^{2}},\\&\mathbb{N}\mathbb{u}=\frac{1}{k_{f}(T_{1}-T_{2})}(-rk_{hnf}(T_{z})_{z=0}),\quad \mathbb{S}\mathbb{h}=\frac{1}{D_{f}(C_{1}-C_{2})}(-rD_{hnf} (C_{z})_{z=0}), \end{aligned} \end{aligned}$$

In order to obtain non-dimensional physical quantities we use Eq. (17) in Eq. (24) to get following form

25 $$\begin{aligned} \begin{aligned} Re^{-1/2}_{x}\mathbb {C}_{f}=\frac{\sqrt{F''(0)^{2}+G'(0)^{2}}}{(1-\varphi _{1})^{2.5}(1-\varphi _{2})^{2.5}},\quad Re^{1/2}_{x}\mathbb{N}\mathbb{u}=-\chi _{4}\theta '(0),\quad Re^{1/2}_{x}Sh=-\chi _{6}\phi '(0). \end{aligned} \end{aligned}$$

### Entropy generation and Bejan number

The local entropy generation of an axially symmetric hybrid nanofluid is^53,55,56^

26 $$\begin{aligned} \begin{aligned} \mathfrak {S}_{gen}=\underbrace{ \frac{k_{hnf}}{T_m^2}(\nabla T)^2}_{\text {Thermal Ir-reversibility}}+\underbrace{\frac{\mu _{hnf}}{T_m}\left[ \frac{1}{k_0}(u^2)+\Phi \right] }_{\text {Fluid Friction Ir-reversibility}}+\underbrace{\frac{1}{T_m}[(\mathcal {J}-\mathcal {Q}\mathcal {V}).(\mathcal {E}+\mathcal {V} \times \mathcal {B})]}_{\text {Joule Dissipation Ir-reversibility}}, \end{aligned} \end{aligned}$$

here

27 $$\begin{aligned} \begin{aligned} (\nabla T)^2=\left[ \left( \frac{\partial T}{\partial r}\right) ^2+\left( \frac{\partial T}{\partial z}\right) ^2\right] ,\\ \Phi =2\left[ \left( \frac{\partial u}{\partial r}\right) ^2+\frac{u^2}{r^2}+\left( \frac{\partial w}{\partial z}\right) ^2\right] +\left( \frac{\partial v}{\partial z}\right) ^2+\left( \frac{\partial w}{\partial r}+\frac{\partial u}{\partial z}\right) ^2+\left[ r\frac{\partial }{\partial r}\left( \frac{v}{r}\right) \right] ^2,\\ \mathcal {J}=\sigma (\mathcal {E}+\mathcal {V}\times \mathcal {B}). \end{aligned} \end{aligned}$$

Now, $T_m$ is the mean temperature between the disks, $k_0$ is the porous medium permeability, $\Phi$ is the viscous dissipation term, $\mathcal {J}$ presents the current density, $\mathcal {Q}$ is the electric charge, $\mathcal {V}$ is the velocity vector and $\mathcal {E}$ is the electric force per unit charge. In Eqs. (26), (27), we assume that $\mathcal {E}$ is much smaller when compared to the cross product $\mathcal {V}\times \mathcal {B}$. Moreover, we assume that the product $\mathcal {Q}\mathcal {V}$ is also negligible in comparison to $\mathcal {J}$. By applying the above assumptions and using Eq. (27) in Eq. (26), we obtain the final local entropy expression as

28 $$\begin{aligned} \begin{aligned} \mathfrak {S}_{gen}=\frac{k_{hnf}}{T_m^2}\left( \frac{\partial T}{\partial z}\right) ^2+\frac{\mu _{hnf}}{T_m}\left[ \frac{1}{k_0}(u^2)+2\left\{ \left( \frac{\partial u}{\partial r}\right) ^2+\frac{u^2}{r^2}+\left( \frac{\partial w}{\partial z}\right) ^2\right\} \right. \\ \left. +\left( \frac{\partial v}{\partial z}\right) ^2+\left( \frac{\partial u}{\partial z}\right) ^2+\left( \frac{\partial v}{\partial r}-\frac{v}{r}\right) ^2\right] +\frac{\sigma _{hnf}}{T_m}(u^2+v^2)B_0^2, \end{aligned} \end{aligned}$$

We use the similarity transforms from Eq. (17) in above Eq. (28) to obtain the non-dimensional expression of the entropy generation given below

29 $$\begin{aligned} \begin{aligned} \mathcal {N}_g=\alpha \theta '^2+Br Re\left( 12 F'^2+\frac{F'^2}{Da}\right) +Br(G'^2+F''^2)+M\frac{\chi _{1}}{\chi _{2}}Br(G^2+F'^2), \end{aligned} \end{aligned}$$

where

30 $$\begin{aligned} \begin{aligned} \mathcal {N}_g=\frac{\mathfrak {S}_{gen}\nu _f T_m}{k_{hnf}\Omega \nabla T},\, \alpha =\frac{\nabla T}{T_m},\, Br=\frac{\mu _{hnf}\Omega ^2 r^2}{k_{hnf}\nabla T (1-bt)^2},\, Re=\nu _f\frac{\Omega }{r^2}, \end{aligned} \end{aligned}$$

here $\mathcal {N}_g$ is th entropy generation rate, $\alpha$ is the dimensionless temperature difference, *Br* is the Brinkman number and *Re* the Reynolds number.

Entropy generation and optimization is much important for scientist. One such number, which is much important in understanding physical aspects of irreversibility is Bejan number. It is the ratio of entropy generation through thermal irreversibility to the total entropy. Bejan number in this study is as follows

31 $$\begin{aligned} \begin{aligned} Be=\frac{\alpha \theta '^2}{\mathcal {N}_g}. \end{aligned} \end{aligned}$$

We observe that Bejan number lies in domain [0,1]. When $Be=1$ the entropy generation is dominated by the thermal irreversibility effects and for $Be=1/2$ heat transfer effects equal to the sum of fluid friction and Joule dissipation irreversibility.

## Proposed methodology with convergence analysis

In order to present basic methodology we consider following system as

32 $$\begin{aligned} \begin{aligned} \mathcal {L}_{i}[\Upsilon _{i}(\eta )]+\mathcal {N}_{i}[\Upsilon _{i}(\eta )]-g_{i}(\eta )=0,\\ B_{i}\left( \Upsilon _{i},\frac{d^{n}\Upsilon _{i}}{d\eta ^{n}}\right) =0, \end{aligned} \end{aligned}$$

where *i* is the index such that $i=1(1)4$ while $\Upsilon _{i}$ and $g_{i}$ are unknown and known functions. $\eta$ is the independent variable.

Firstly, construct homotopies as^57,58^

33 $$\begin{aligned} \begin{aligned} (1-\breve{q})\mathcal {L}_{i}[\Upsilon _{i}(\eta ;\breve{q})-\Upsilon _{i0}(\eta )]=\hbar _{i}\breve{q}H(\eta )\mathcal {N}_{i}[\Upsilon _{i}(\eta ;\breve{q})], \end{aligned} \end{aligned}$$

where

34 $$\begin{aligned} \Upsilon _{i}(\eta ;0)=\Upsilon _{i0}(\eta ), \end{aligned}$$

and

35 $$\begin{aligned} \Upsilon _{i}(\eta ;1)=\Upsilon _{i}(\eta ), \end{aligned}$$

Now we expand the unknown function $\Upsilon _{i}(\eta ;q)$ into a power series of $\breve{q}$,

36 $$\begin{aligned} \Upsilon _{i}(\eta ;\breve{q})=\Upsilon _{i0}(\eta )+\sum _{m=1}^{+\infty }\Upsilon _{im}(\eta )\breve{q}^{m}, \end{aligned}$$

and

37 $$\begin{aligned} \Upsilon _{im}(\eta )=\left. \frac{1}{m!}\frac{\partial \Upsilon _{i}(\eta ;\breve{q})}{\partial \breve{q}^{m}}\right| _{\breve{q}=0}, \end{aligned}$$

In order to obtain the $m\text {-}th$ order deformation, we use Eq. (36) in Eq. (33) and get the following

38 $$\begin{aligned} \mathcal {L}_{i}[\Upsilon _{im}(\eta )-\varkappa _{im}\Upsilon _{i(m-1)}(\eta )]=\hbar _{i}H_{i}(\eta )\mathcal {Q}_{im}(\eta ), \end{aligned}$$

here

39 $$\begin{aligned} \mathcal {Q}_{im}(\eta )=\frac{1}{(m-1)!}\left. \frac{\partial ^{m-1}\mathcal {N}_{i}[\Upsilon _{i}(\eta ;\breve{q})]}{\partial \breve{q}^{m-1}}\right| _{q=0}, \end{aligned}$$

and

40 $$\begin{aligned} \varkappa _{im}={\left\{ \begin{array}{ll} 0, \text {when }m\le 1,\\ 1, \text {when }m\ge 2, \end{array}\right. } \end{aligned}$$

In case of Eqs. (18)–(21), the initial guesses and linear operators are as follows

41 $$\begin{aligned} \begin{aligned} \mathcal {L}_{F}=\frac{\partial ^3 F}{\partial \eta ^3},\, \mathcal {L}_{G}=\frac{\partial ^2 G}{\partial \eta ^2},\, \mathcal {L}_{\theta }=\frac{\partial ^2 \theta }{\partial \eta ^2},\, \mathcal {L}_{\phi }=\frac{\partial ^2 \phi }{\partial \eta ^2},\\ F_{0}(\eta )=\frac{\zeta _{1} x^2+\zeta _{2} x^2+2 \zeta _{1} x+2 \zeta _{2} \delta x}{2 (\delta +1)},\, G_{0}(\eta )=1-\eta ,\, \phi _{0}(\eta )=1-\eta , \, \theta _{0}(\eta )=1-\eta . \end{aligned} \end{aligned}$$

The *m*-*th* order series form solutions can be obtained by adding initial guesses with the special functions obtained through Eq. (38) for $i=1,2,3$ and 4, respectively.

### Convergence analysis of proposed methodology

**Figure 2:**
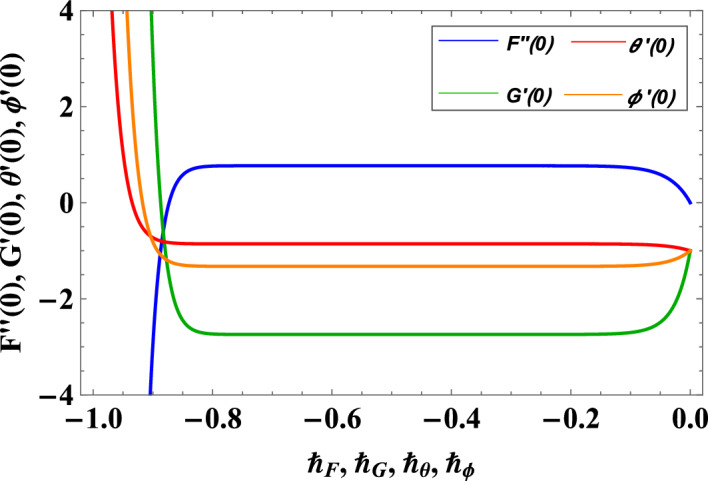
$\hbar$-curves for velocity, temperature and concentration profiles.

**Figure 3:**
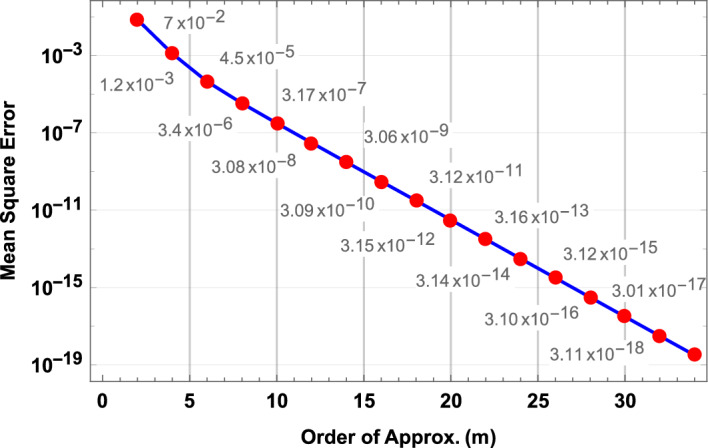
Convergence of HAM solution.

The convergence of series solution is determined through $\hbar$-curves of the system. $\hbar$-curves provide interval of convergence graphically. The combined $\hbar$-curves for $F,G,\theta$ and $\phi$ are plotted at $28^{th}$ order in Fig. 2. Moreover, after fixing values of fluid parameters and $\hbar$ (chosen from the region of convergence), series form solutions are presented in Table 3. It is noted that solutions converged at $23^{rd}$, $29^{th}$ till $51^{st}$ order, all correct up to 6 decimal places. Convergence is also shown in Fig. 3 where means square errors are plotted against order of approximation. Furthermore, in Table 4 HAM solutions at $4^{th}$ order are also compared with numerical solutions obtained through Runge-Kutta 4th order method for different values of $\eta$ and it is noted that both solutions are same upto fourth decimal.

**Table 3:** Convergent HAM solutions at different orders of approximation when *M*=2.3, $\mathbb {U}$=1.2, *Pr*=0.1, $\mathcal {S}_c$=0.9, $\zeta _{1}$=1.7, $\zeta _{2}$=1.2, *Da*=2.1, $H_{s}$=1.5, $\lambda$=1.1, $n_{1}$=3.0, $n_{2}$=3.0 and $\hbar _{i}$=-0.5.

Order	$Sol_{F}$	$-Sol_{G}$	$-Sol_{\theta }$	$-Sol_{\phi }$
3	0.669179	2.65476	0.874397	1.29304
8	0.765734	2.73974	0.856625	1.32333
11	0.768473	2.74143	0.856092	1.32368
17	0.768862	2.74163	0.856014	1.3237
23	0.768868	2.74163	0.856013	1.3237
29	0.768868	2.74163	0.856013	1.3237
35	0.768868	2.74163	0.856013	1.3237
42	0.768868	2.74163	0.856013	1.3237
51	0.768868	2.74163	0.856013	1.3237

**Table 4:** Validation of HAM results with RK4 when *M*=2.3, $\mathbb {U}$=1.2, *Pr*=0.1, $\mathcal {S}_c$=0.9, $\zeta _{1}$=1.7, $\zeta _{2}$=1.2, *Da*=2.1, $H_{s}$=1.5, $\lambda$=1.1, $n_{1}$=3.0, $n_{2}$=3.0 and $\hbar _{i}$=-0.5.

$\eta$	$Sol_{F}$		$Sol_{G}$		$Sol_{\theta }$		$Sol_{\phi }$	
	HAM	RK4	HAM	RK4	HAM	RK4	HAM	RK4
0.0	0.	0.	1.	1.	1.	1.	1.	1.
0.2	0.2769	0.2769	0.5634	0.5634	0.8178	0.8178	0.7387	0.7387
0.4	0.5462	0.5461	0.2987	0.2986	0.6188	0.6188	0.4969	0.4969
0.6	0.8077	0.8077	0.1434	0.1434	0.4108	0.4108	0.2893	0.2893
0.8	1.0609	1.0609	0.0536	0.0536	0.2021	0.2021	0.1235	0.1235
1.0	0.	0.	0.	0.	0.	0.	0.	0.

## Results and discussion

The hybrid nanofluid flow between two rotating disks is simulated for various fluid parameters and physical interpretations are drawn in this section for velocity, temperature and concentration profiles. Parameters of physical interest like entropy generation, Bejan number, skin friction, Nusselt number and Sherwood number are also discussed in detail.

### Velocity profile

**Figure 4:**
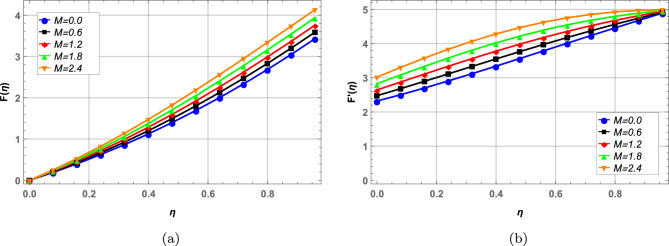
Effect of magnetic field on radial and axial velocity.

**Figure 5:**
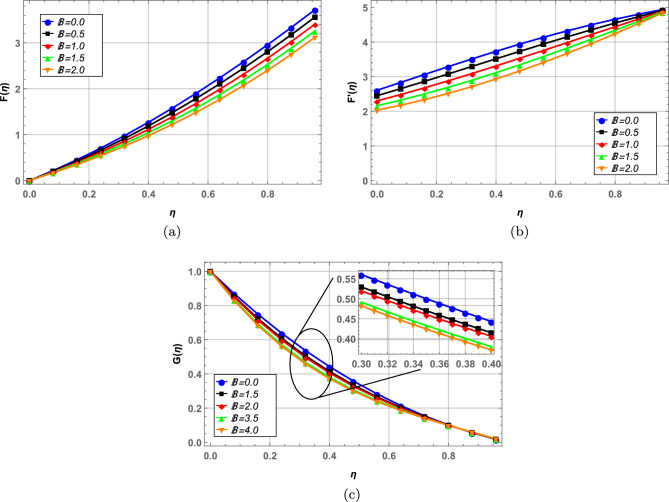
Radial, axial and tangential velocity against unsteady parameter.

**Figure 6:**
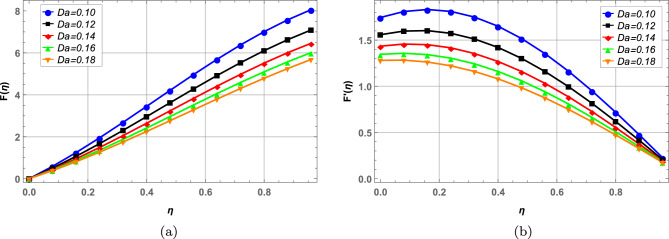
Effect of Darcy number on radial and axial velocity.

**Figure 7:**
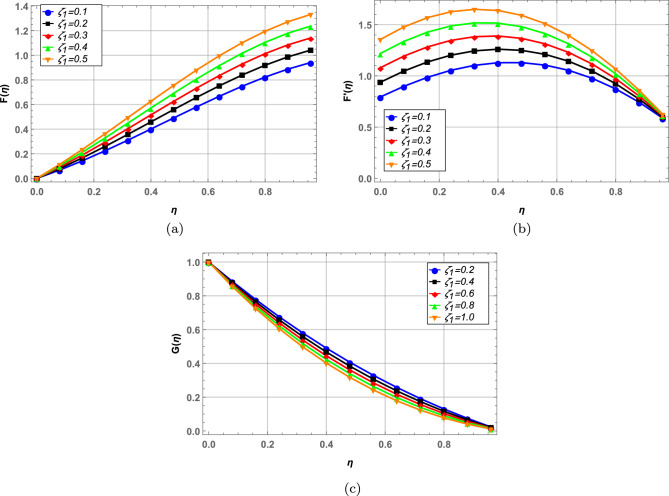
Radial, axial and tangential velocity against stretching at lower disk.

**Figure 8:**
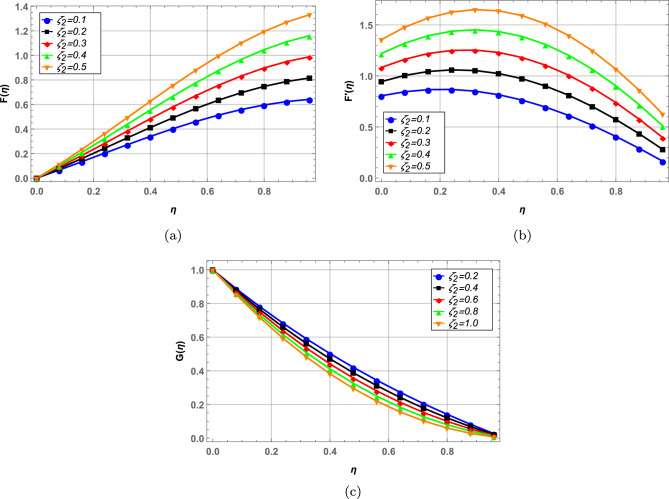
Radial, axial and tangential velocity against stretching at upper disk.

**Figure 9:**
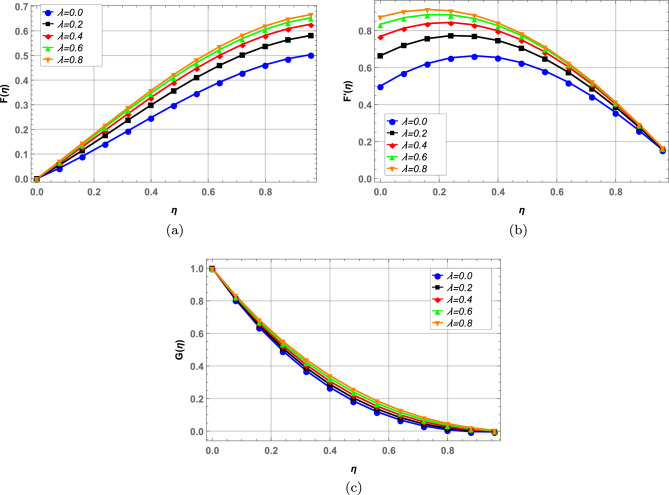
Effect of slip parameter on velocity profile.

Radial, axial and tangential velocities are presented against pertinent fluid parameters in Figs. 4, 5, 6, 7, 8, 9. Increase in magnetic interaction parameter increases the radial and axial velocities in Fig. 4a and b. Magnetic parameter being inversely related to the fluid density, increases the blood flow as base fluid density decreases. The unsteady parameter $\mathbb {B}$ decreases, the radial tangential and axial velocity in Fig. 5a–c, respectively. Increase in unsteady parameter decreases the disk rotation, resulting in decreased fluid flow. In Figs. 6a and b, increase in the Darcy number *Da* decreases the radial and axial velocity.Larger Darcy number results in increased viscous forces among fluid layers that causes resistance to fluid flow in radial and axial directions. Increase in stretching parameters $\zeta _{1}$ and $\zeta _{2}$ increases fluid velocity in radial and axial direction, whereas a decrease in fluid flow is observed tangentially (see Figs. 7 and 8). Increase in both $\zeta _{1}$ and $\zeta _{2}$ is increasing the stretching motion of disk in *u*-direction and decreasing the rotation of disk in $\theta$-direction which offers increased flow in radially and axially while decreasing it in tangential direction. In Fig. 9, increase in slip parameter $\lambda$, increases the fluid velocity in all directions as resistance to flow decreases.

### Temperature profile

**Figure 10:**
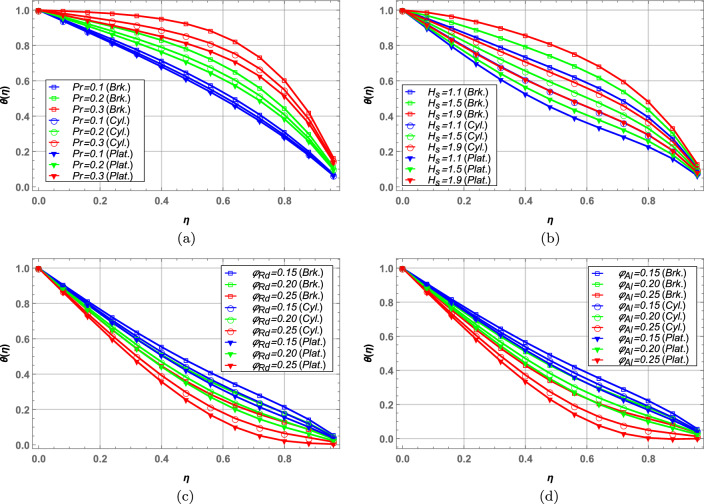
Temperature profile for brick, cylindrical and platelet shaped nanoparticles.

Change in fluid temperature is observed in Figs. 10a–d in case of brick, cylinder and platelet shaped nanoparticles comparatively. As seen in Fig. 10a, higher values of Prandtl number *Pr* increases the fluid temperature due to elevated thermal diffusivity. Increase in heat source $H_{s}$ increases the temperature of nanofluid in Fig. 10b. Moreover, increase in volume fraction of radium nanoparticles $\varphi _{Rd}$ and alumina nanoparticles $\varphi _{Al}$ decreases the blood temperature in Fig. 10c and d. It is observed that brick shape case shown the highest while platelet shape case shown the lowest temperature throughout the paper.

### Concentration profile

**Figure 11:**
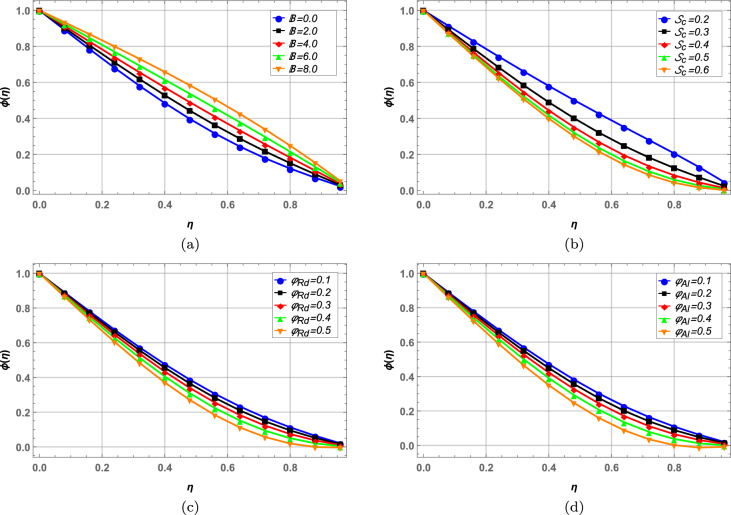
Concentration profile against various fluid parameters.

Concentration of blood nanofluid against unsteady parameter $\mathbb {B}$, Schmidt number $\mathcal {S}_{c}$ and nanoparticle volume fractions $\varphi _{Rd}$ and $\varphi _{Al}$ is presented in Fig. 11. In Fig. 11a, higher values of unsteady parameter increases the blood concentration. Increase in Schmidt number $\mathcal {S}_{c}$ decreases the concentration of blood hybrid nanofluid in Fig. 11b. Schmidt number is the ratio between momentum diffusivity an mass diffusivity in a fluid flow. So, higher values of Schmidt number correspond to more diffusion through momentum, resulting in lower concentration of the nanofluid. Increasing the volume fractions of both radium and alumina decreases the blood concentration in Figs. 11c and d, respectively.

### Entropy generation and Bejan number

**Figure 12:**
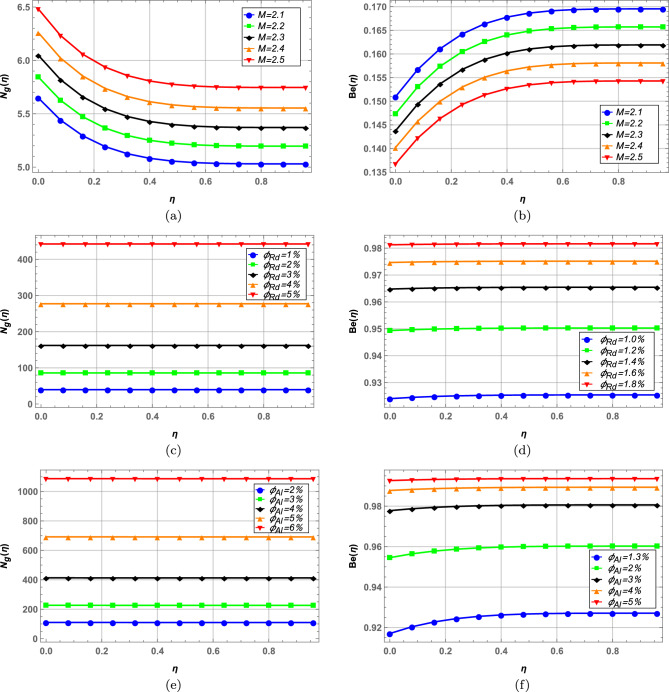
Entropy and Bejan number against magnetic field and nanoparticle volume fractions.

**Figure 13:**
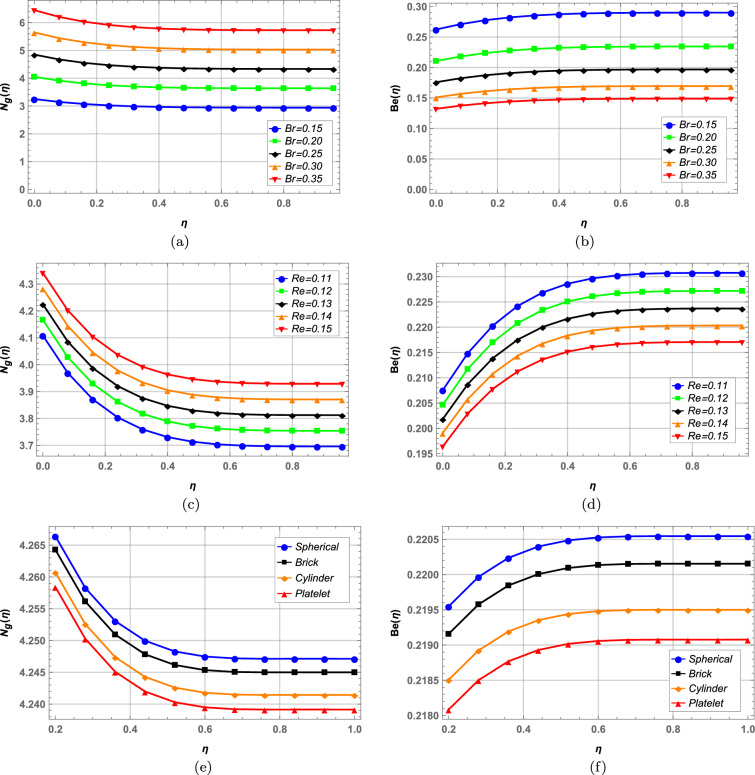
Entropy and Bejan number against Brinkman number, Reynolds number and nanoparticle shapes.

Entropy and Bejan number are plotted side by side in Figs. 12, 13 and entropy optimization is numerically analyzed in Table 5. Increase in magnetic interaction parameter *M* in Fig. 12a increases entropy and Bejan number shows inverse behavior in Fig. b. As *M* increases the resistance in fluid flow increases due to the Lorentz forces and disorderedness of system increases. Increase in volume fraction of radium and alumina increases entropy while Bejan number behaves oppositely (see Figs. 12c–f). Higher Brinkman number *Br* increases entropy as seen in Fig. 13a and Bejan number is decreased in contrast in Fig. 13b. In c increase in Reynolds number *Re* elevates entropy generation as higher values of Reynolds number results in more turbulent flow due to which system becomes more disordered. Bejan number on the other hand shows opposite behavior in Fig. 13d. It is evident from Fig. 13e and f that spherical shaped nanoparticles offer highest entropy while platelet shaped nanoparticles offer lowest entropy in hybrid nanofluid flow. Bejan number in Fig. 13f depicts similar behavior. In Table 5 numerically minimized entropy is calculated for variable values of $\hbar$ and volume fractions $\varphi _{1}$ and $\varphi _{2}$ along with optimal values of all other fluid parameters required to achieve minimum entropy. In case of $\hbar$, minimum entropy is achieved when $\hbar =-0.6$. Overall, the minimum entropy in blood hybrid nanofluid is obtained to be zero when volume fraction of radium nanoparticles is $2\%$ and that of alumina is $1\%$ while keeping $\hbar =-0.6$.

**Table 5:** Minimization of Entropy Generation.

Variable			Optimized fluid parameters												
$\hbar$	$\varphi _{Rd}$	$\varphi _{Al}$	*M*	$\mathbb {U}$	$-Pr$	*Sc*	$-Hs$	$\zeta _{1}$	$-\zeta _{2}$	*Da*	$\lambda$	*Br*	*Re*	$\alpha$	$N_{g}$-min
−0.3	0.1%	0.1%	5.32	4.82	0.19	1.44	0.97	0.61	0.56	0.80	0.96	0.36	0.26	0.27	2.3 $E{-33}$
−0.5			−11.98	−7.98	0.99	1.43	1.28	0.51	0.24	1.51	0.99	0.52	0.14	0.61	5.5 $E{-31}$
−0.6			5.15	4.52	0.18	1.35	0.72	0.46	0.52	0.96	0.71	0.	0.07	−0.50	4.8$E{-35}$
−1.0			3.36	1.96	0.64	0.25	0.85	0.07	0.01	0.35	0.40	0.53	−0.20	0.21	7.5$E{-31}$
−0.6	2%	1%	−6.92	−4.81	0.15	0.44	1.05	0.37	0.29	0.31	0.87	0.07	−0.51	0.21	4.9$E{-31}$
	3%		0.33	0.37	0.11	0.76	1.01	0.46	0.27	0.15	0.77	0.03	−0.55	0.17	0.
	4%		−1.55	−0.76	0.15	0.73	1.04	0.44	0.24	0.11	0.74	0.04	−0.57	0.14	2.2$E{-32}$
	5%		−3.18	−1.71	0.16	0.74	1.02	0.43	0.24	0.12	0.70	0.03	−0.53	0.15	6.1$E{-33}$
	1%	2%	1.07	1.41	0.17	1.39	0.62	0.45	0.43	1.02	0.73	−0.12	−0.01	0.48	1.4$E{-31}$
		3%	1.09	1.43	0.17	1.39	0.62	0.45	0.43	1.01	0.73	−0.12	−0.01	0.48	1.1$E{-30}$
		4%	−5.20	−2.64	0.13	1.38	0.60	0.45	0.37	0.97	0.76	−0.12	−0.01	0.50	3.2$E{-33}$
		5%	−5.04	−2.54	0.13	1.38	0.60	0.45	0.37	0.97	0.76	−0.12	−0.01	0.49	2.9$E{-32}$

### Skin friction, Nusselt and Sherwood number

**Figure 14:**
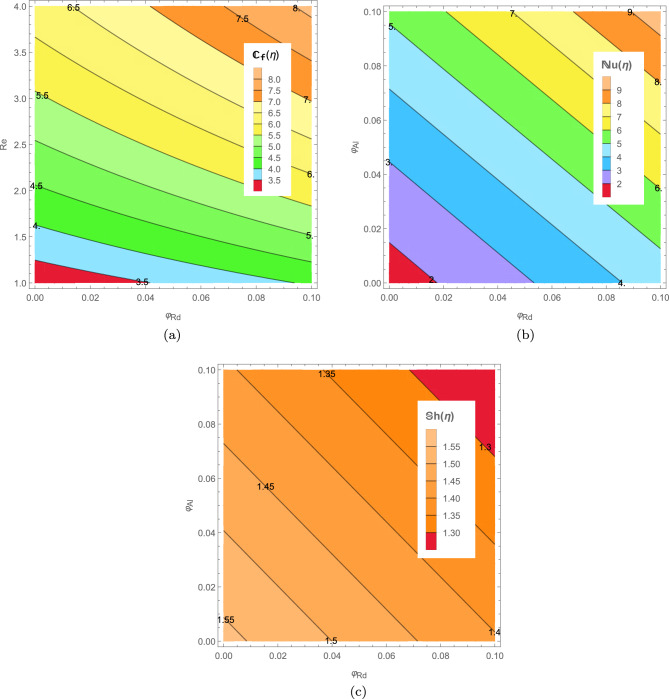
Analysis of skin friction, Nusselt number and Sherwood number through contours.

**Figure 15:**
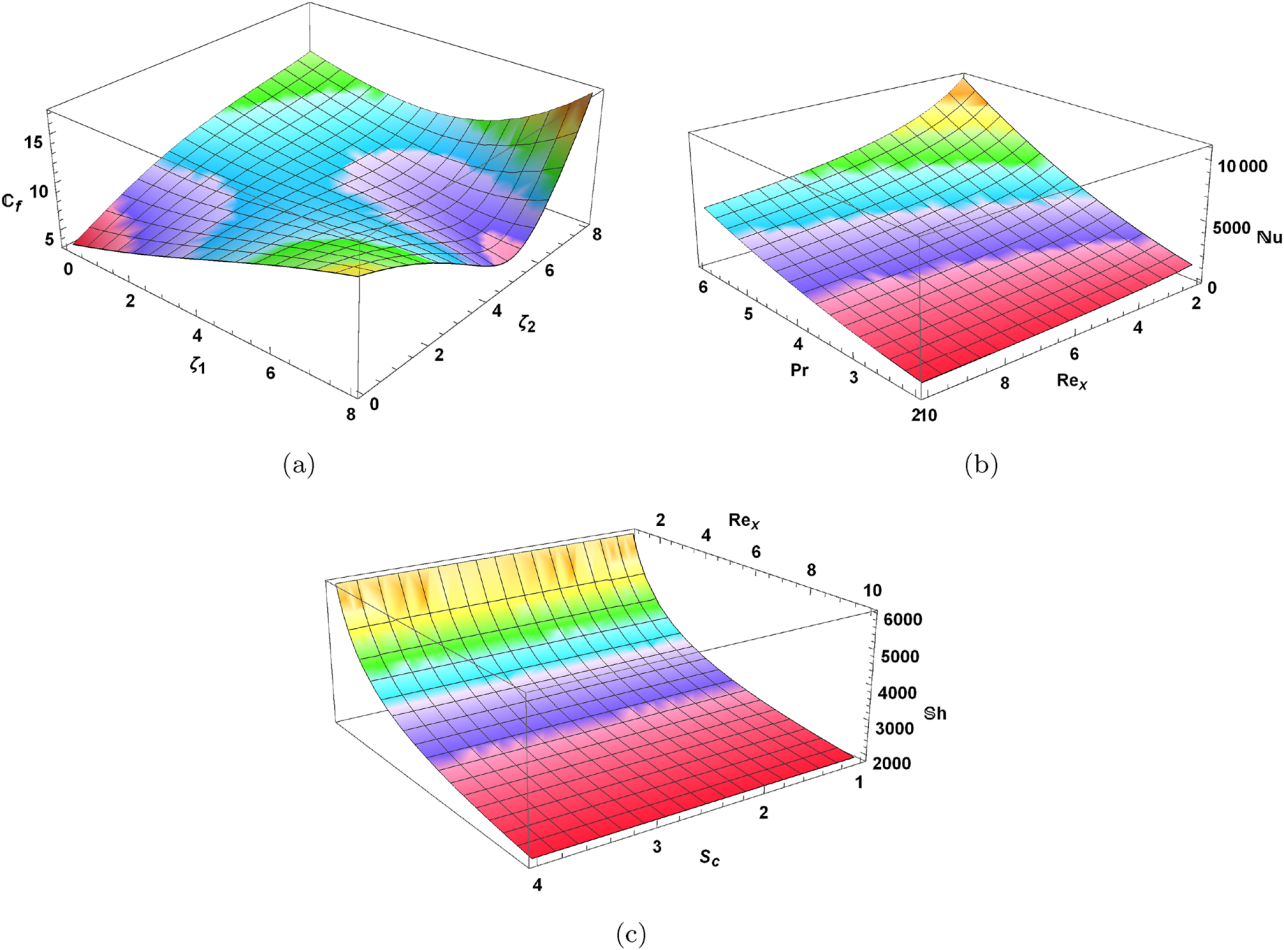
3D analysis of skin friction, Nusselt number and Sherwood number.

**Figure 16:**
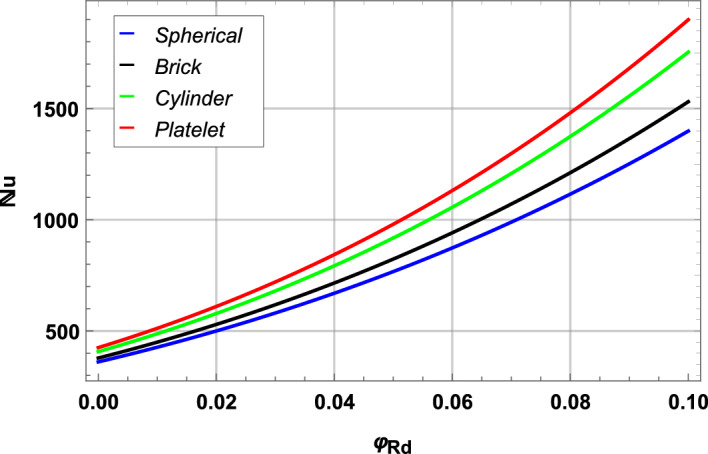
Heat transfer profile for spherical, brick, cylindrical and platelet shaped nanoparticles.

In Figs. 14 and 15 we present skin friction, heat and mass transfer in form of contours and 3D plots. Moreover, comparative heat transfer rate is also presented in a 2D plot for different shapes of nanoparticles in Fig. 16. Skin friction of hybrid blood nanofluid increases with increase in Reynolds number *Re* and volume fraction of radium nanoparticles $\varphi _{Rd}$ as depicted in Fig. 14a. It is evident from the contour plot that increase in skin friction with respect to *Re* is much more significant when compared to increase caused by volume fraction $\varphi _{Rd}$. Increase in nanoparticle concentration of radium and alumina results in elevated heat transfer rate in Fig. 14b. This increase in heat transfer is more prominent when alumina nanoparticles $\varphi _{Al}$ are increased as compared to radium nanoparticles. The reason of higher heat transfer caused by alumina is due to higher thermal conductivity of $Al_{2}O_{3}$ than *Rd* as given in Table 2. Mass transfer rate in Fig. 14c decreases as $\varphi _{Rd}$ and $\varphi _{Al}$ increases. Both stretching parameters $\zeta _{1}$ and $\zeta _{2}$ are plotted simultaneously against skin friction in a 3D plot presented in Fig. 15a. Increase in $\zeta _{1}$ increases the skin friction of nanofluid with the disk surface while $\zeta _{2}$ first decreases the skin friction till $\zeta _{2}=5.4$ and then increases onward. In Fig. 15b Reynolds number *Re* and Prandtl number *Pr* are presented against Nusselt number. Increase in *Re* decreases the heat transfer rate while increase in *Pr* increases the heat transfer rate in blood nanofluid. Maximum heat transfer is seen at a corner peak with highest *Pr* and lowest *Re*. Increase in *Re* and Schmidt number $\mathcal {S}_{c}$ is depicted in Fig. 15c for Sherwood number. Higher values of *Re* decreases the rate of mass transfer substantially. Furthermore, rate of heat transfer for various nanoparticle shapes is shown in Fig. 16. Platelet shaped nanoparticles offer highest heat transfer rate while spherical shape of nanoparticles offer lowest rate of heat transfer.

## Conclusion

The objective of current manuscript is modeling and computation of entropy generation and optimization in hybrid nanofluid with different nanoparticle shape factors. The base fluid in current study is blood while the two nanoparticles are radium *Rd* and alumina $Al_{2}O_{3}$. The blood flow is simulated between two rotating and stretching disk with porous medium and slip effects at the boundaries. The system of non-linear ordinary differential equations of this fluid model is solved through a novel semi analytical approach namely, homotopy analysis method (HAM). The series form solution obtained through HAM is tested for convergence and error analysis. The series solution is convergent from the 23rd order of approximation onward and the errors are as low as $10^{-19}$ at 35th order of approximation. Velocity, temperature and concentration profiles are analyzed through 2D plots. Entropy generation is analyzed numerically and graphically for various parameters. Moreover, skin friction, Nusselt number and Sherwood number are studied through contour and 3D plots for better understanding and presentation purpose. Major results of the investigation are:

- Fluid velocity decreases in all directions (radial, axial and tangential) with increase in unsteady parameter $\mathbb {B}$ and Darcy number *Da*.
- Both stretching parameters $\zeta _{1}$ and $\zeta _{2}$ elevate the velocity profile in radial and axial directions whereas tangential velocity shows opposite results.
- Increase in slip parameter $\lambda$ increases velocity in axial, radial and tangential directions.
- Temperature increases with higher values of Prandtl number *Pr* and heat source $H_{s}$ while opposite behavior is observed with increase in $\varphi _{Rd}$ and $\varphi _{Al}$.
- Overall temperature of the fluid is highest in case of brick shaped nanoparticles whereas platelet shape nanoparticles result in lowest fluid temperature.
- Concentration of blood hybrid nanofluid increases with higher values of unsteady parameter $\mathbb {B}$ while decrease in fluid concentration is recorded with increase in Schmidt number $\mathcal {S}_{c}$ and nanoparticle volume fractions.
- Entropy generation increases with increase in magnetic parameter *M*, volume fractions $\varphi _{Rd}$, $\varphi _{Al}$, Brinkman number *Br* and Reynolds number *Re* whereas Bejan number behaves in contrast.
- Spherical shaped nanoparticles result in highest entropy while the platelet shaped nanoparticles offer lowest entropy.
- The most optimal value of entropy is obtained to be zero when $\hbar =-0.6$, $\varphi _{Rd}=2\%$ and $\varphi _{Al}=1\%$.
- Skin friction of nanofluid with wall elevates with higher values of Reynolds number *Re*, volume fraction $\varphi _{Rd}$, stretching parameters $\zeta _{1}$ and $\zeta _{2}$.
- Mass transfer decreases with increase in both volume fractions $\varphi _{Rd}$, $\varphi _{Al}$ and Reynolds number *Re*.
- Heat transfer in the fluid is highest in case of platelet shaped nanoparticles and lowest in case of spherical shaped nanoparticles.

This study can be further carried out in future by fractional modeling for various nanofluid models in both Buongiorno and two phase cases.

## Data Availability

All data generated or analyzed during this study are included in this article.

## List of symbols

$u,\,v,\,w$: Radial, tangential and axial velocity
$$\nu$$: Kinematic viscosity (m$$^{2}s^{-1}$$)
$$\rho$$: Density (kgm$$^{-3}$$)
$(\rho C_{p})$: Specific heat (*J*/*kgK*)
$Q_{s}$: Heat source/sink
*C*: Fluid concentration
$$U_{slip}$$: Slip velocity
$$\Omega$$: Disk rotation (s$$^{-1}$$)
$$\chi _{i}$$: Nanofluid parameters
$$\lambda$$: Slip parameter
$$\mathbb {B}$$: Unsteady parameter
$H_{s}$: Heat source/sink parameter
$$\zeta _{1},\,\zeta _{2}$$: Stretching parameters
$\mathcal {N}_{g}$: Entropy generation rate
*Br*: Brinkman number
*Re*: Reynolds number
$\alpha$: Dimensionless temperature difference
$t,\,r,\,z$: Temporal, radial and axial coordinates
$\sigma$: Electric conductivity (Sm$^{-1}$)
$B_{0}^{2}$: Magnetic field strength (Am$^{-1}$)
*D*: Thermal diffusivity (m$$^{2}s^{-1}$$)
*T*: Fluid temperature
$U_{w1},\,U_{w2}$: Velocity at lower and upper disk
*L*: Slip distance (m)
*b*: Constant (s$$^{-1}$$)
*Da*: Darcy number
$$\mathcal {S}_{c}$$: Schmidt number
*Pr*: Prandtl number
*M*: Magnetic interaction parameter
$$\varphi$$: Volume fraction

## Subscripts

‘*hnf*’: Hybrid nanofluid
‘*f*’: Base fluid
‘*Rd*’, ’*Al*’: Radium and alumina quantity
